# Chronic Eosinophilic Leukemia Positive for FIP1L1-PDGFRa

**DOI:** 10.7759/cureus.79548

**Published:** 2025-02-24

**Authors:** Steven J Haydon, Danielle E Ross, Stephen Caucci, Shane Clark, Borys Hrinczenko

**Affiliations:** 1 Internal Medicine, Mclaren Greater Lansing, Lansing, USA; 2 College of Osteopathic Medicine, Michigan State University, East Lansing, USA; 3 Hematology and Medical Oncology, McLaren Greater Lansing, Lansing, USA; 4 College of Human Medicine, Michigan State University, East Lansing, USA

**Keywords:** chronic leukemia, eosinophilic leukemia, fip1l1-pdgfra, imatinib therapy, thombocytopenia

## Abstract

*FIP1L1-PDGFRα-*positive chronic eosinophilic leukemia (CEL) is a rare subtype of myeloproliferative neoplasm characterized by organ damage caused by eosinophilic granules containing cytokines and humoral factors. These include interleukin-5 (IL-5), interleukin-4 (IL-4), and interleukin-13 (IL-13). We present a case study of a 71-year-old male who initially presented with cough, fever, night sweats, and weight loss. Fluorescence in situ hybridization (FISH) and cytogenetic testing confirmed that the patient had *FIP1L1-PDGFRα-*positive chronic eosinophilic leukemia. Treatment with imatinib and prednisone led to a rapid decrease in the eosinophil count without further damage from eosinophilic infiltration. Our patient has tolerated therapy with occasional lightheadedness that he attributed to external factors, and he continues to remain in remission. This case study aims to contribute to the existing knowledge regarding the discovery and treatment of this disease.

## Introduction

Chronic eosinophilic leukemia (CEL) is a rare condition characterized by elevated levels of eosinophils, which can lead to organ damage due to the release of cytokines and other humoral factors [1]. The humoral factors causing damage include interleukin-5 (IL-5), interleukin-4 (IL-4), and interleukin-13 (IL-13), which are involved in the upregulation and activation of eosinophils, leading to tissue damage through the release of toxic granular proteins such as eosinophil cationic protein, eosinophil-derived neurotoxin, eosinophil peroxidase, and major basic protein [2]. Due to its rarity and difficulty in diagnosing, apart from other forms of hypereosinophilia, its true incidence remains unknown [1,3,4].

CEL falls under the umbrella of hypereosinophilic syndromes (HES). The WHO 5th edition classification for eosinophilia-associated neoplasms requires persistent eosinophilia (≥1.5 x 10⁹/L) for at least four weeks, with reactive and genetic causes excluded. Diagnosis necessitates both clonality evidence and abnormal bone marrow morphology while ruling out other myeloid and lymphoid neoplasms. Blasts must be <20%, and specific cytogenetic aberrations (e.g., tyrosine kinase gene fusions, *FLT3*, *JAK2*, *ETV6::ABL1*, and AML-related translocations) must be absent [1]. Differentiation from idiopathic hypereosinophilic syndrome requires genetic testing in the form of FISH and cytogenetic studies. Genetic testing has shown that mutations in genes such as *PDGFRA*, *PDGFRB*, *FGFR1*, *JAK2*, *ABL1*, or* FLT3* are often associated with CEL [4]. Patients with specific genetic translocations, such as *FIP1L1-PDGFRα*, may experience gastrointestinal, skin, or cardiopulmonary symptoms, or may be asymptomatic [5]. Due to its rarity, the prevalence of symptomatology remains unknown. In this case study, a 71-year-old man with coronary artery disease presented with night sweats and a dry cough, leading to a diagnosis of *CHIC2* deletion with *FIP1L1-PDGFRα* translocation.

## Case presentation

We present a 71-year-old man with a history of coronary artery disease. He was referred to our hematology-oncology clinic in March 2022 for evaluation of thrombocytopenia observed during a recent hospitalization at an outside facility. His social history included working in a religious field. He denied any current tobacco, alcohol, or illicit drug use. He had a history of tobacco use where he smoked two packs per day for three years. Family history was significant for the mother having breast cancer and the father having lung cancer.

Records from this hospitalization indicated that he had visited the emergency room 11 days before his appointment with complaints of dizziness and nausea. Workup during his evaluation consisted of a computed tomography (CT) scan of his brain/head which revealed no acute abnormalities. He was initially treated with meclizine for his dizziness thought to be consistent with benign paroxysmal positional vertigo with insignificant symptom improvement. During his emergency department visit, his blood work revealed thrombocytopenia, leukocytosis, and normal hemoglobin. An elevated absolute eosinophil count was also noted at 5.9x10^3^/uL (0.0-0.6x10^3^/uL). He reported experiencing easy bruising and skin changes, described as “petechial lesions”, for several weeks prior to this evaluation. He also reported drenching night sweats multiple times per week, a dry cough, and unintentional weight loss over the past five months. Physical exam was largely unremarkable aside from splenomegaly and some minor ecchymosis on his extremities. Prior records were reviewed with findings significant for a platelet count of 100x10^3^/uL two years before this hospitalization. However, at the time of admission, his platelet count was noted to decrease to 49x10^3^/uL (150-400x10^3^/uL). He was admitted to the hospital for further evaluation due to his dizziness and thrombocytopenia. These symptoms raised concerns for hematologic malignancy, an infectious and/or allergic etiology, an autoimmune condition, or lung diseases. Given this hypereosinophilia, thrombocytopenia, and constitutional symptoms, attention was turned toward working up for malignancy, while ruling out other etiologies.

Hematology was consulted, and an initial workup was conducted, including HIV and viral hepatitis testing, both of which returned negative. Cytomegalovirus and Epstein-Barr virus testing both indicated past infection. Serum protein electrophoresis and free light chain analysis were unremarkable. Stool studies assessing for the presence of ova and parasites were negative. No coagulopathies or abnormalities were noted on multiple complete metabolic panels. An abdominal ultrasound demonstrated splenomegaly. He was ultimately discharged after 4 days with a plan to follow up with hematology in the outpatient setting.

During his initial appointment at the hematology-oncology clinic, 11 days post-admission, a follow-up complete blood count (CBC) was obtained (Table 1). Additionally, an antinuclear antibody (ANA) screen yielded negative results. It was recommended that the patient return for additional blood work and consult with an allergist regarding their chronic cough with eosinophilia. He subsequently followed up three months later in June 2022. During this visit, a repeat CBC revealed the findings demonstrated in table 1. He mentioned that he had been seen by an allergist since his previous appointment and no significant allergies were identified. Due to the worsening thrombocytopenia and persistent eosinophilia, it was decided that the patient would undergo a bone marrow biopsy within the next week to further characterize any abnormalities in the *PDGFRA*, *PDGFRB*, *FGFR1*, and *PCM1-JAK2* genes.

**Table 1:** Lab values throughout initial diagnosis and treatment with Imatinib WBC: white blood cell count, HgB: hemoglobin, PLT: platelet count, EOS: eosinophil count

Date	WBC (10^3^/uL)	HgB (g/dL)	PLT (10^3^/uL)	EOS (10^3^/uL)
Reference Ranges	04-12	12.6-16.5	150-400	0-0.72
August, 2020	5.1	15	100	0.1
March, 2022 (initial ED visit)	10.8	13.4	44	5.92
March, 2022 (initial clinic visit)	11.2	14.1	40.9	5.83
June, 2022	7.5	12.9	28	4.32
August, 2022 (initiation of imatinib)	6.7	14.1	34	1.9
August, 2022 (1 week of imatinib treatment)	3.14	12.9	62	0.02
July, 2023 (1 year of imatinib treatment)	6.05	15.6	78	0.7
August, 2024 (2 years of imatinib treatment)	5.73	15.5	72	0.5

**Figure 1:**
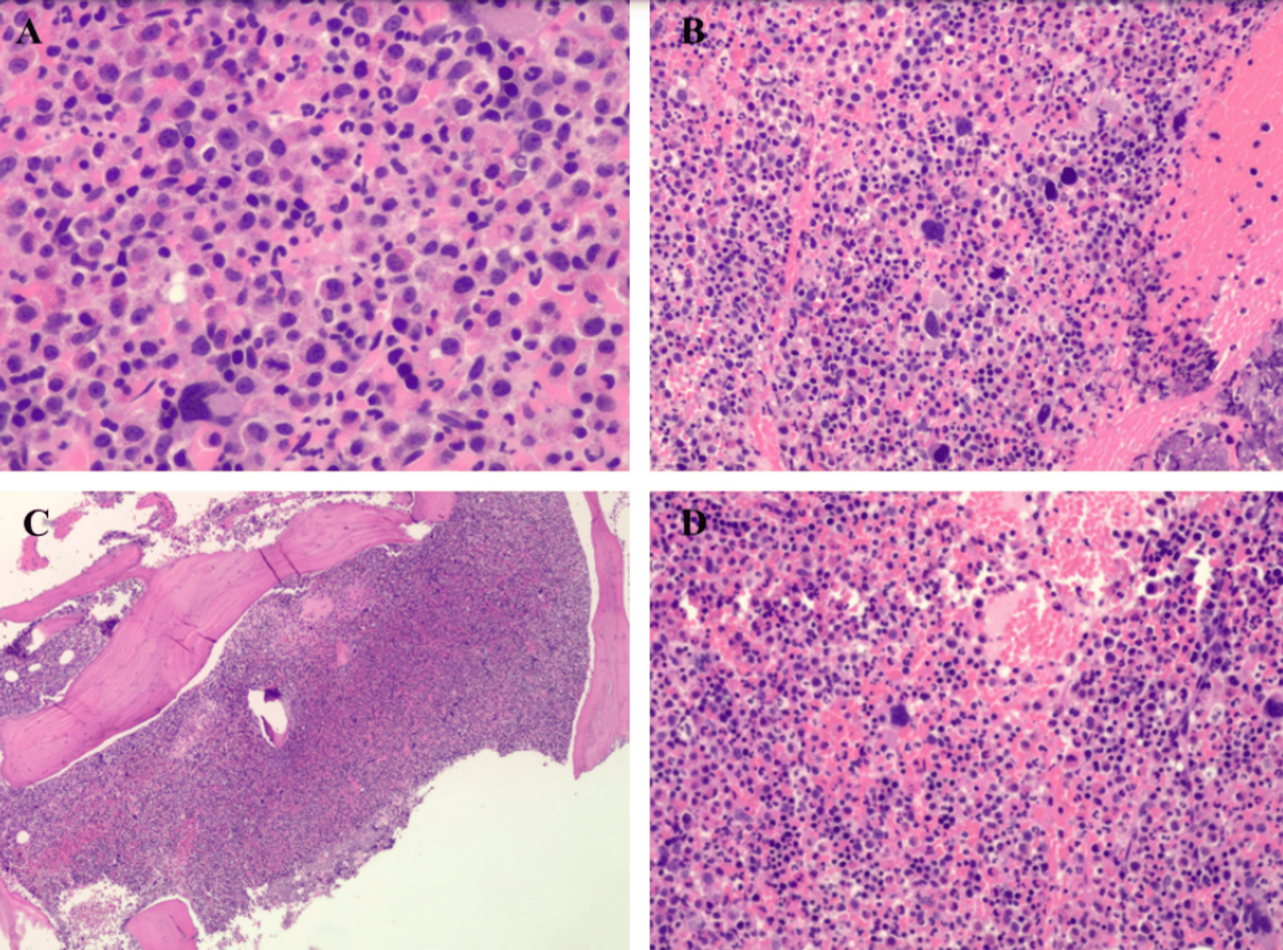
Bone marrow biopsy showing hematoxylin and eosin (H&E) stains A) Marrow is hypercellular with increased eosinophilic elements including eosinophilic myelocytes. B) Many megakaryocytes are abnormal with chromatin condensation and cloud like nucleus. C) Hypercellular myeloid dominant marrow with increased eosinophilic precursors. D) Hypercellular marrow redemonstrated.

The results from the biopsy indicated a significantly high number of myeloid cells in the bone marrow, with an abundance of eosinophilic precursors, suggesting a possible myeloid neoplasm. Histology images obtained from the bone marrow biopsy are shown in figure 1. Further testing, such as FISH and cytogenetic testing, confirmed the diagnosis of chronic eosinophilic leukemia with a *CHIC2* deletion and *FIP1L1-PDGFRα* translocation, showing 60% of abnormal cells.

The case was presented at a multidisciplinary tumor board, where a treatment plan was determined. The treatment regimen consisted of prednisone 70 mg daily (1mg/kg/d), followed by a steroid taper and initiation of Imatinib 100mg daily after completing the steroid course.

Prior to starting imatinib, a thorough transthoracic echocardiogram revealed normal cardiac function with some left ventricular apical thickening. Further investigations with cardiac magnetic resonance imaging (cMRI) did not reveal any apical thickening, thus ruling out hypertrophic cardiomyopathy. In consideration of potential eosinophilic infiltration, a prednisone loading dose was necessary before starting Imatinib. Although there were no previous echocardiograms available for comparison, imatinib was eventually initiated in early August 2022 after delays caused by the patient's COVID-19 diagnosis.

Following the initiation of imatinib therapy, our patient's absolute eosinophil count reached 0.02 K/mcl within one week (normal range: 0.0-0.06 x10^3^/uL). This was an expected result. Subsequently, his eosinophil counts have consistently remained within normal limits. The patient has reported generally tolerating imatinib well, with only a few missed doses due to transient dizziness, which he attributed to external factors. He has maintained a daily dose of 100mg of imatinib since the beginning of treatment.

## Discussion

Chronic eosinophilic leukemia (CEL) is a rare myeloid neoplasm with an unknown incidence, according to the SEER database, due to its rarity in the population [4]. When evaluating patients with high eosinophil levels, it is important to consider potential secondary causes such as infection, allergies, lung disease, and autoimmune conditions [3]. Once secondary causes have been ruled out, it is necessary to conduct tests including CBC, B12 levels, ESR, CRP, and tryptase levels [6]. In this case, a serum tryptase level was not ordered for the patient. In hindsight, tryptase levels could have been ordered, but this did not affect diagnosis and treatment. Research has shown a link between elevated tryptase levels and CEL, as well as a correlation between male sex, cardiac and pulmonary symptoms, and *FIP1L1-PDGFRα*-positive CEL [7]. There is no current data that provides an odds ratio or prevalence rate that compares tryptase levels directly to CEL. However, a tryptase level of >20ng/dl warrants investigation to differentiate between mastocytosis and CEL [8]. The increased number of eosinophils in the bone marrow raised suspicion of malignancy. These findings suggest the importance of considering FISH analysis and other diagnostic techniques early on.

In cases where the cause of eosinophilia is not clear, it is recommended to proceed with karyotype and FISH analysis. Specifically, the presence of a *FIP1L1-PDGFRα* fusion gene is linked to a tyrosine kinase mutation that may stimulate eosinophilic proliferation [4]. Studies suggest that around 5-15% of individuals with CEL carry this mutation [9]. Lambert et al recommend using FISH and reverse transcription polymerase chain reaction (RT-PCR) together as the primary diagnostic methods to minimize the risk of false negatives [10]. This mutation is more common in males and is linked to symptoms, such as splenomegaly, fever, weight loss, elevated B12 levels, and cough, which align with our patients' presentation [11]. These symptoms and lab values can correlate to other pathologies, such as other myeloid malignancies or idiopathic HES. FISH and cytogenetic studies are crucial in reaching a diagnosis of CEL [2]. Additionally, cases of endocardial involvement and cardiac hypertrophy due to eosinophilic infiltration are predominantly seen in patients with the *FIP1L1-PDGFRα* mutation [5]. Further research may also be indicated to evaluate the frequency of cardiac involvement in relation to *FIP1L1-PDGFRα* mutations.

**Figure 2:**
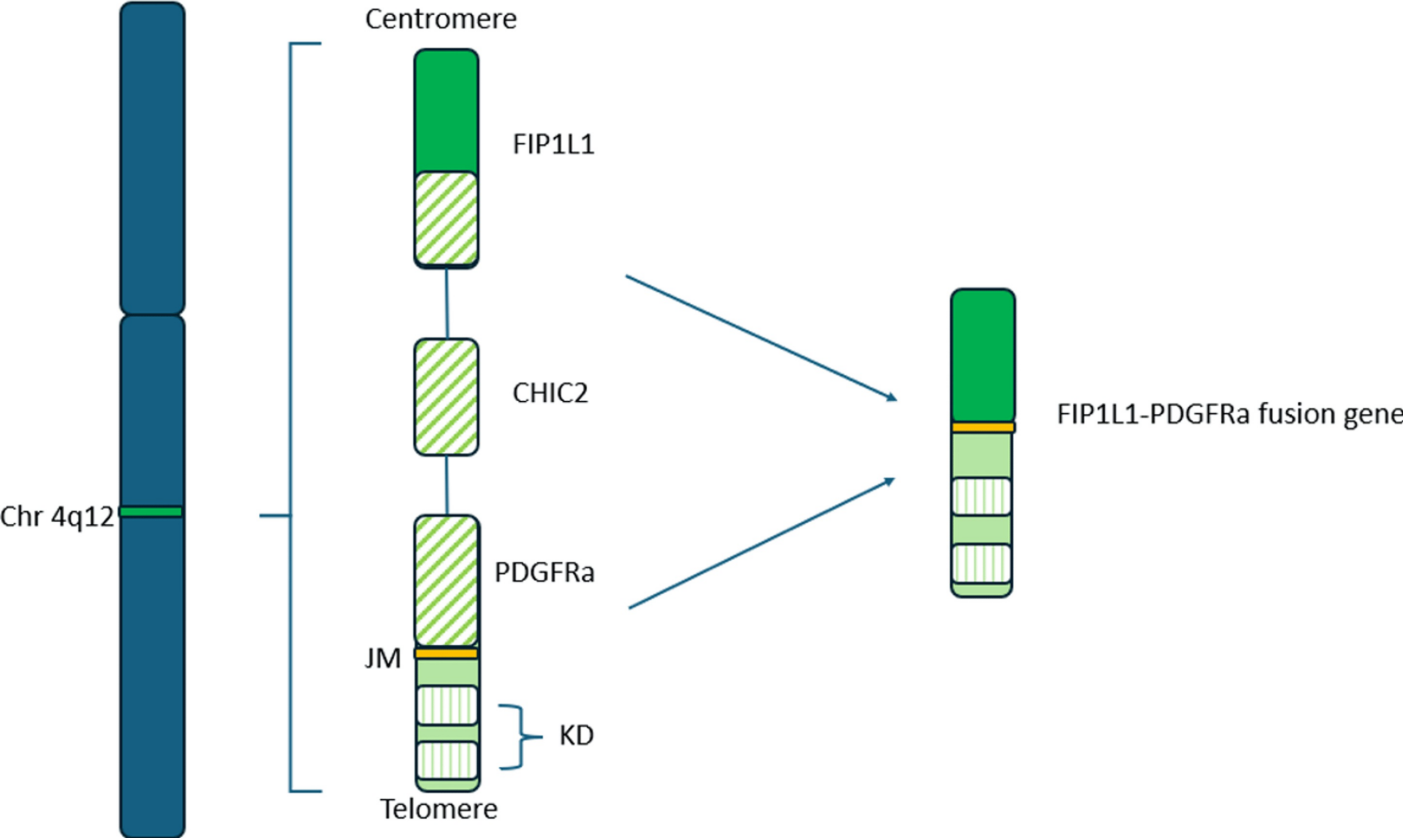
Generation of the FIP1L1-PDGFRa fusion gene with deletion of CHIC2 through del(4)(q12). This allows for constitutive action of kinase domains on PDGFRa. Image credits: Steven Haydon Chr: chromosome; JM: juxtamembrane domain; KD: kinase domain

The *FIP1L1-PDGFRα* fusion gene is the result of the deletion on Chromosome 4q12 [12]. While oligomerization of *FIP1L1-PDGFRα* has not been demonstrated, there is evidence of a disruption of the juxtamembrane of *PDGFRα* leading to constitutive activation of its tyrosine kinase activity [12]. This constitutive action occurs when there is a partial or complete removal of the juxtamembrane domain of *PDGFRα* (Figure 2) [12]. FIP1L1 plays a critical role as a promoter and translation start for the fusion gene, influencing the stability of the protein [12]. The exact pathways involved in stimulating eosinophils downstream from the fusion protein are not fully understood but may involve signaling pathways such as phosphoinositol 3-kinase, *ERK 1/2*, and *STAT*. A study in mice using only the *FIP1L1-PDGFRα* mutation did not result in high levels of eosinophilia, suggesting that overexpression of IL-5 may contribute to the disease seen in CEL [7].

Research has shown that patients with CEL who have the *FIP1L1-PDGFRα* fusion gene may respond well to treatment with the tyrosine kinase inhibitor, imatinib [11,13]. This mutation leads to the activation of tyrosine kinase. Based on the National Comprehensive Cancer Network (NCCN) guidelines, the recommended imatinib dosage for CEL patients with the* FIP1L1-PDGFRa* fusion gene is 100- 400mg daily. If there is suspicion of cardiac involvement, a short course of steroids should be given before starting imatinib to prevent acute heart failure and death [5]. Typically, treatment includes using a 1mg/kg/d dosing with sufficient depletion of eosinophils, as seen in our patient.

Due to the limited research on this condition, the optimal dosage and duration of imatinib treatment are not well-established. A study conducted by Klion et al. involved gradually reducing the imatinib dosage for five CEL patients with the *FIP1L1-PDGFRα* fusion gene from 300-400mg daily to 100mg daily. If there was no molecular evidence of the fusion gene after six months of treatment at 100 mg daily, imatinib was discontinued. However, all patients in the study experienced molecular relapse after dose reduction and discontinuation of therapy [14]. This raises the question of whether imatinib therapy needs to be lifelong or if there is a course for discontinuation.

Although there is potential for achieving molecular remission with low doses of imatinib, careful consideration should be given before discontinuing therapy. It may be important to follow established guidelines like those for chronic myeloid leukemia, such as monitoring *BCR-ABL1* quantitative assays at three, six, and 12 months of therapy initiation [15]. Future studies could explore strategies for discontinuing tyrosine kinase inhibitor therapy to reduce recurrence rates in CEL and offer alternative options in cases of resistance. Historical data demonstrates that patients diagnosed with CEL prior to imatinib therapy had a mean survival time of 9 months [16]. Overall survival data in patients with *FIP1L1-PDGFRα*-positive CEL receiving imatinib is approximately 89% at ten years [8].

## Conclusions

In summary, we present a 71-year-old man presenting with nonspecific symptoms of cough, weight loss, and night sweats who was ultimately diagnosed with *FIP1L1-PDGFRα* mutation-positive chronic eosinophilic leukemia. These findings illustrate the need for prompt diagnosis. This case highlights the importance of extensive workup and evaluation in patients presenting with similar non-specific symptoms and elevations in absolute eosinophilia, as well as a demonstration of the significant effect of daily imatinib therapy in the restoration of normal activities of daily living. In addition, prednisone treatment before imatinib therapy was imperative to avoid complications from his potential cardiac involvement. He, like others with the *FIP1L1-PDGFRα* mutation, showed a swift response to imatinib therapy that has been sustained for over 24 months, as evidenced by his absolute eosinophil counts. Given the rarity of this form of leukemia, further data regarding overall survival, appropriateness of TKI discontinuation, and second-line therapy is needed.
